# India Rubber Cones for Polishing Vulcanized Rubber

**Published:** 1866-02

**Authors:** 


### INDIA RUBBER CONES FOR POLISHING VULCANIZED RUBBER.

Before I began to use vulcanized rubber as a base for artificial teeth I had seen several of my professional friends using pine and cork cones, with sand paper attached to them by a ring of metal forced over the sand paper till it came near the base of the cone, which was attached to a small lathe, for the purpose of smoothing and polishing vulcanized rubber. That plan of procedure was always somewhat unsatisfactory to me, and I had received from a friend a piece of cast-away India rubber, which had been used on a railroad car, and laid it away for some future use, but at the time I did not know what I would want it for; but, as the pine wood cones did not last, nor work well with me, nor the cork cones, which were what are called jug corks by dealers in corks, I concluded to try how a gum elastic cone would work, and selected as sound a portion of the old rubber as I could get, cut out a piece as smooth as I could with my pocket knife; (but it was not very easy to work smooth, and dulled the edge of the knife very badly.) The largest one I made was about two and a quarter inches long, one and a quarter inches in diameter at the base, tapering to about half an inch in diameter at the point, which was rather to large in case a man had but one, but I find it advantageous to have two; one about one-third the size above mentioned. I made three sizes, but would recommend but two for that purpose. Having used these three cones a great deal, I find they answer the purpose much better than anything else I have tried. They wear out very slowly, and their elasticity permits pulverized pumice stone, and tripoli, moistened or wet with water, to adhere sufficiently to answer the purpose very well. I found some difficulty in getting a hole in the centre to insert the tapering screw that belongs to and accompanies the *hand and foot lathe*, but I succeeded by the use of a tine of a rat-tail file, which I heated a little, and by that means bored and burned it until I could screw it on and make it fast to the lathe.

If other dentists have not discovered anything better adapted to the purpose, I would respectfully suggest to the manufacturers of gum elastic toys and wares to prepare a mould with a conical central piece in it, so as to form a small hole in the cone, extending about two-thirds of the length, and manufacture a sufficient number of them to supply all the dentists who use vulcanized rubber as a base for artificial teeth; but if there are any other means better adapted to the purpose, I would be pleased to know of it, as I have been seeking the best means of performing the duties of a dentist for many years, and have rejoiced at many of the improvements which have been made, and gladly lend my feeble aid in the advancement of our profession.—*Dental Times*.

